# Experimental Study on Durability Improvement of Fly Ash Concrete with Durability Improving Admixture

**DOI:** 10.1155/2014/818103

**Published:** 2014-06-09

**Authors:** Hong-zhu Quan, Hideo Kasami

**Affiliations:** ^1^School of Architectural Engineering, Qingdao Agricultural University, Qingdao 266109, China; ^2^Japan Association for Building Research Promotion, Tokyo 108-0014, Japan

## Abstract

In order to improve the durability of fly ash concrete, a series of experimental studies are carried out, where durability improving admixture is used to reduce drying shrinkage and improve freezing-thawing resistance. The effects of durability improving admixture, air content, water-binder ratio, and fly ash replacement ratio on the performance of fly ash concrete are discussed in this paper. The results show that by using durability improving admixture in nonair-entraining fly ash concrete, the compressive strength of fly ash concrete can be improved by 10%–20%, and the drying shrinkage is reduced by 60%. Carbonation resistance of concrete is roughly proportional to water-cement ratio regardless of water-binder ratio and fly ash replacement ratio. For the specimens cured in air for 2 weeks, the freezing-thawing resistance is improved. In addition, by making use of durability improving admixture, it is easier to control the air content and make fly ash concrete into nonair-entraining one. The quality of fly ash concrete is thereby optimized.

## 1. Introduction

Coal is widely used in China as fuel for power plants because of its dependability and economy. In 2009, more than 375 million tons of fly ash was generated from coal-fired power plants in China. How to effectively use fly ash is still a major social problem. In order to construct recycling society, researchers have found many ways to make efficient use of fly ash in civil engineering field [1–10], but in fact the use of fly ash in building construction is still limited. The main reasons are as follows. (1) The quality of fly ash is unstable because the quality of fly ash changes greatly with that of coals [11, 12]; (2) there is lower early age compressive strength of concrete due to lower activity of fly ash [13, 14]; (3) pozzolanic reaction of fly ash leads to decreased pH value of concrete, which results in a poor carbonation resistance [15–17]. In addition, in order to ensure the resistance of freezing-thawing action, it is necessary to entrain a certain amount of air in the concrete. However, air-entraining admixture is adsorbed by the unburned activated carbon in fly ash which reduces air-entraining capability of fly ash concrete [18, 19]. Therefore, it is the key issue to keep serial and stable air in fly ash concrete. In other words, the air content control of fly ash concrete is difficult.

On the basis of the above mentioned background, this paper focused on the nonair-entraining concrete. The effects of durability improving admixture, air content, water-binder ratio, and fly ash replacement ratio on strength development, drying shrinkage, carbonation, and freezing-thawing resistance of fly ash concrete are to be investigated.

## 2. Experiment Overview

### 2.1. Materials Used and Concrete Composition

The characteristics of materials used and the properties of fly ash used are shown in Tables 1 and 2.  Table 3 shows the experiment factors and ratios. Figure 16 shows the interpretation of marks. The mixture proportion and properties of concrete are shown in Table 4.

In this experiment, fly ash (FA) was used to replace the internal mass of cement, and 10 kg/m^3^ of durability improving admixture was added to the final mixture. Air-entraining water reducing admixture was added to all the concrete, accounting for 1% of the weight of binder. In order to regulate fixed air content (4.5%), the standard air-entraining concrete (hereinafter referred to as “ST”) used special air-entraining water reducing admixture. The concrete using durability improving admixture (hereinafter referred to as “D”) became nonair-entraining type concrete, because of strong antifoam reaction in the admixture itself. The nonair-entraining concrete (hereinafter referred to as “PL”) obtained fixed 1% air content by antifoam admixture. The target slump of concrete was 18 cm, and the target air content was 4.5% (ST) and 1.0% (D, PL).

### 2.2. Fabrication and Curing of Test Specimens

Cylindrical specimens, 100 mm in diameter and 200 mm in height, for compression tests were encased in light gauge molds in 3 layers and stripped molds after 24 hours. Then, specimens were cured in water at 20°C until testing ages. Prism specimens were used for drying shrinkage tests, accelerating carbonation tests, and freezing and thawing tests, the dimension of which was 100 mm × 100 mm × 400 mm. The specimens were cast in 2 layers and stripped molds after 24 hours. Then, the specimens for drying shrinkage tests were cured in water at 20°C for 7 days; the specimens for accelerating carbonation tests were cured in water at 20°C until 28 days and stored at air dried condition at 20°C for 28 days; the specimens for freezing-thawing tests were cured in water at 20°C until 28 days.

### 2.3. Test Methods for Hardened Concrete

#### 2.3.1. Compressive Strength Tests

The specimens were tested at the age of 7 days, 28 days, and 91 days in wet condition according to JIS A 1108-2006. Modulus of elasticity tests were measured at the same time of compressive strength tests according to JIS A 1149-2010.

#### 2.3.2. Drying Shrinkage Tests

The specimens were taken from water at 7 days and then stored at 20°C and 60% R.H. in air, and were measured their length change and weight loss for 13 weeks according to JIS A 1129-1-2010.

#### 2.3.3. Accelerating Carbonation Tests

After being moist-cured for 28 days and air-dried for 28 days, the specimens were stored in accelerating carbonation chamber at 20°C, 60% R.H., and 5% CO_2_ concentration according to JIS A 1153-2012. Carbonation depth was determined by phenolphthalein test on sprinkled surface.

#### 2.3.4. Freezing-Thawing Tests

The specimens were subjected to freezing-thawing cycles at the age of 28 days according to ASTM C666/C666M-2003(2008) or JIS A 1148-2010.

## 3. Test Results and Discussions

### 3.1. Compressive Strength

**Figure 1:**
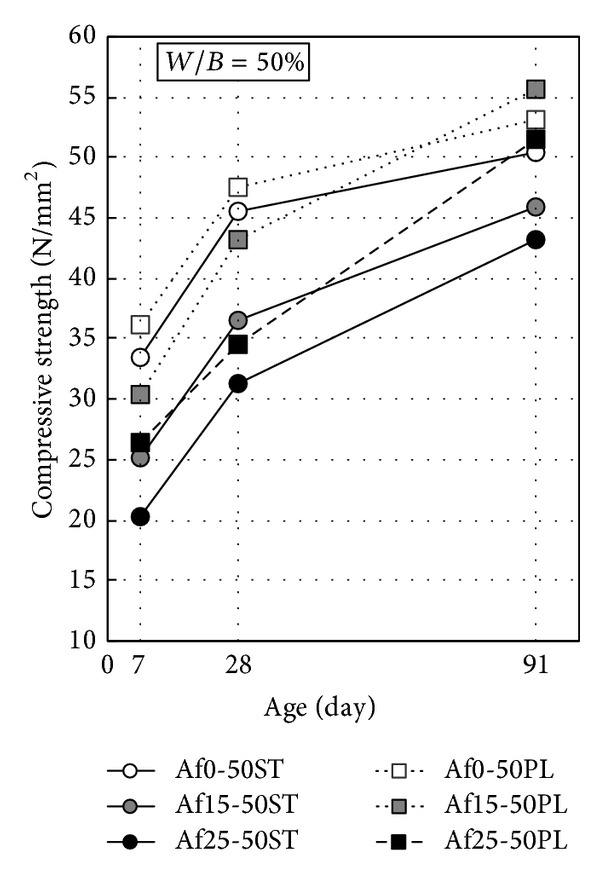
Compressive strength development of concrete (cured in water at 20°C).

**Figure 2:**
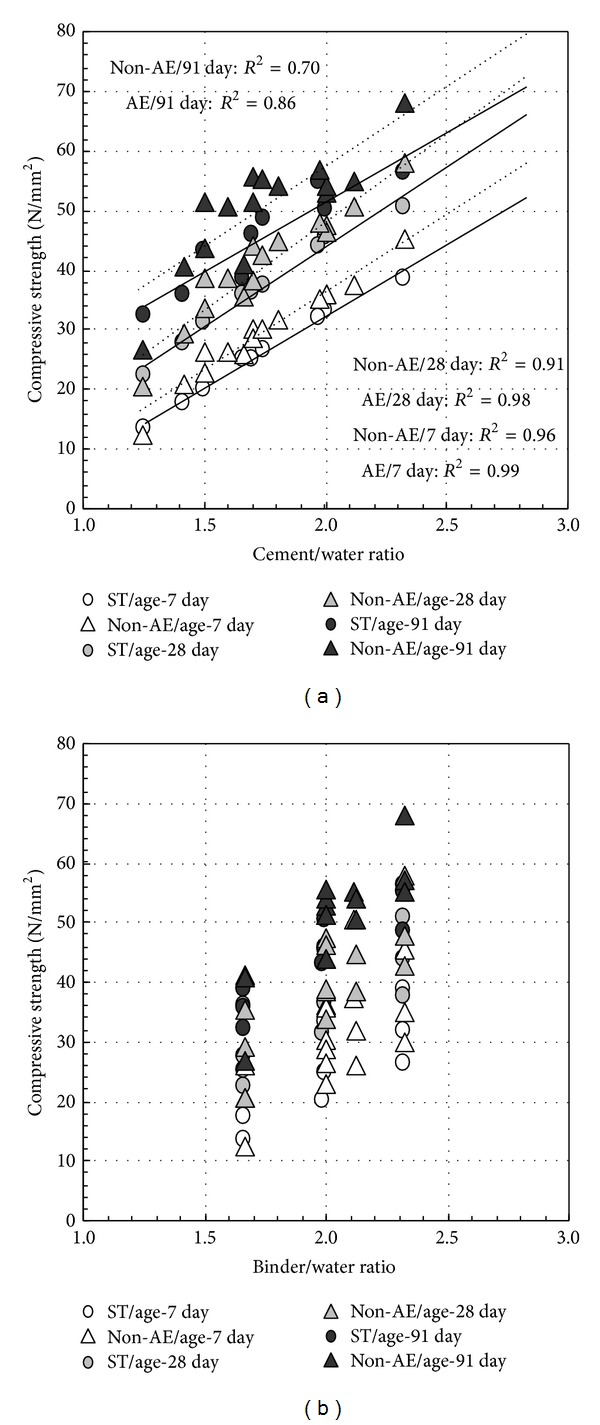
Relationship among cement-water ratio, binder-water ratio, and compressive strength.

Figure 1 shows the compressive strength development of concrete (at *W*/*B* = 50%, cured in water at 20°C). The compressive strength of concrete reduces with the increase of fly ash replacement ratio. This result is not related to water-binder ratio and the presence or absence of durability improving admixture and air content. However, compressive strength growth of concrete is found to be increased with the increase of fly ash replacement ratio after 28 days. The relationship of cement-water ratio and compressive strength and the relationship of binder-water ratio and compressive strength are shown in Figure 2. Since the compressive strength development has no difference between PL and D concrete (nonair-entraining type), both are expressed as an approximate straight line in this figure. Until the age of 28 days, a high correlation is shown between cement-water ratio and compressive strength, regardless of fly ash replacement ratio. After 28 days, however, because the assistance of pozzolanic reaction of fly ash to strength increasing, the correlation between cement-water ratio and compressive strength is poor. Even though there is high correlation between binder-water ratio and compressive strength over a longer period more than 1 year [20], it is unrealistic to evaluate its strength development in short term of 91 days. Also, the effects of air content are as follows: (1) compressive strength of nonair-entraining concrete (D, PL) is 10% higher than air-entraining concrete (ST); (2) to achieve the same strength, the water-cement ratio of nonair-entraining concrete can be increased by around 5%. The correlation between binder-water ratio and compressive strength is higher in the same fly ash replacement ratio. The compressive strength of nonair-entraining concrete (D, PL) is 10%–20% stronger than air-entraining concrete (ST) in the same binder-water ratio depending on the presence or absence of air content (at *W*/*B* = 50% or *W*/*B* = 43%).

### 3.2. Tensile Strength and Modulus of Elasticity

**Figure 3:**
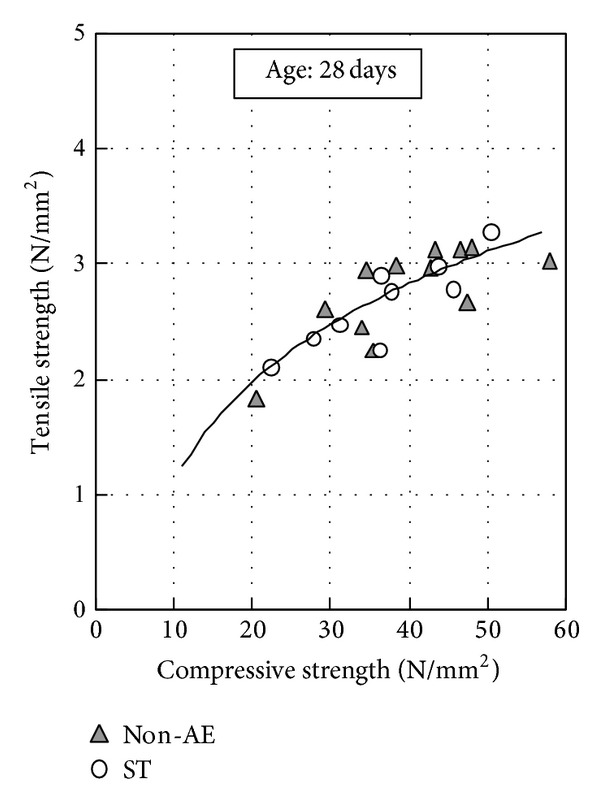
Relationship between compressive strength and tensile strength.

**Figure 4:**
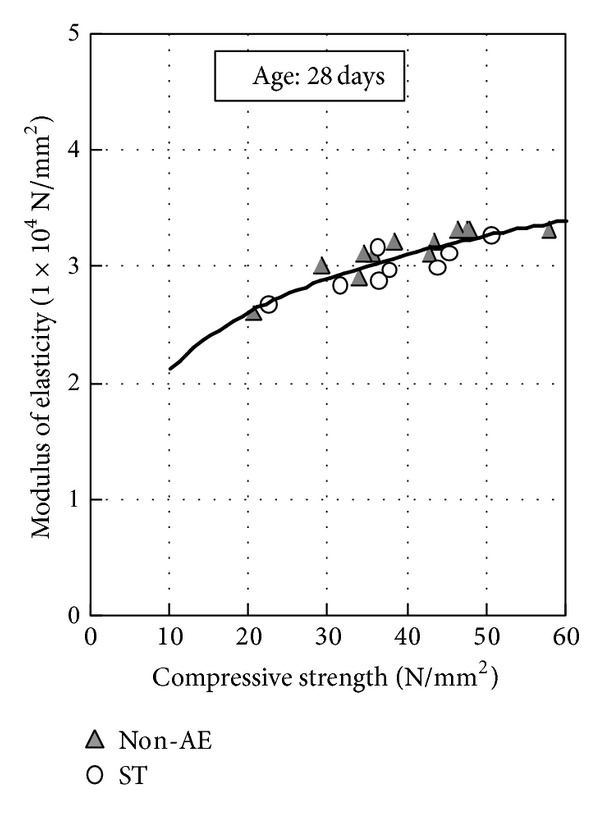
Relationship between compressive strength and modulus of elasticity.

Figure 3 shows the relationship between compressive strength and tensile strength. The values of the 28-day tensile strength cured at about 20°C are in the range of 1/10 to 1/20 in compressive strength. Irrespective of the presence of air content, durability improving admixture, or fly ash replacement ratio, it can be seen that tensile strength is dependent on compressive strength.  Figure 4 shows the relationship between compressive strength and modulus of elasticity. The modulus of elasticity of concrete is approximately 3.1 × 10^4^ N/mm^2^ at 28 days. Modulus of elasticity showed the same tendency as tensile strength.

### 3.3. Drying Shrinkage

**Figure 5:**
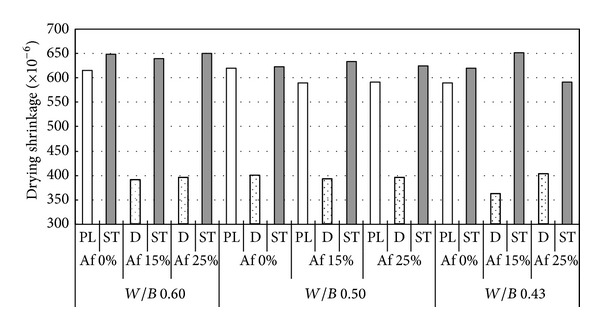
Drying shrinkage of ST, PL, and D concrete at 13 weeks.

**Figure 6:**
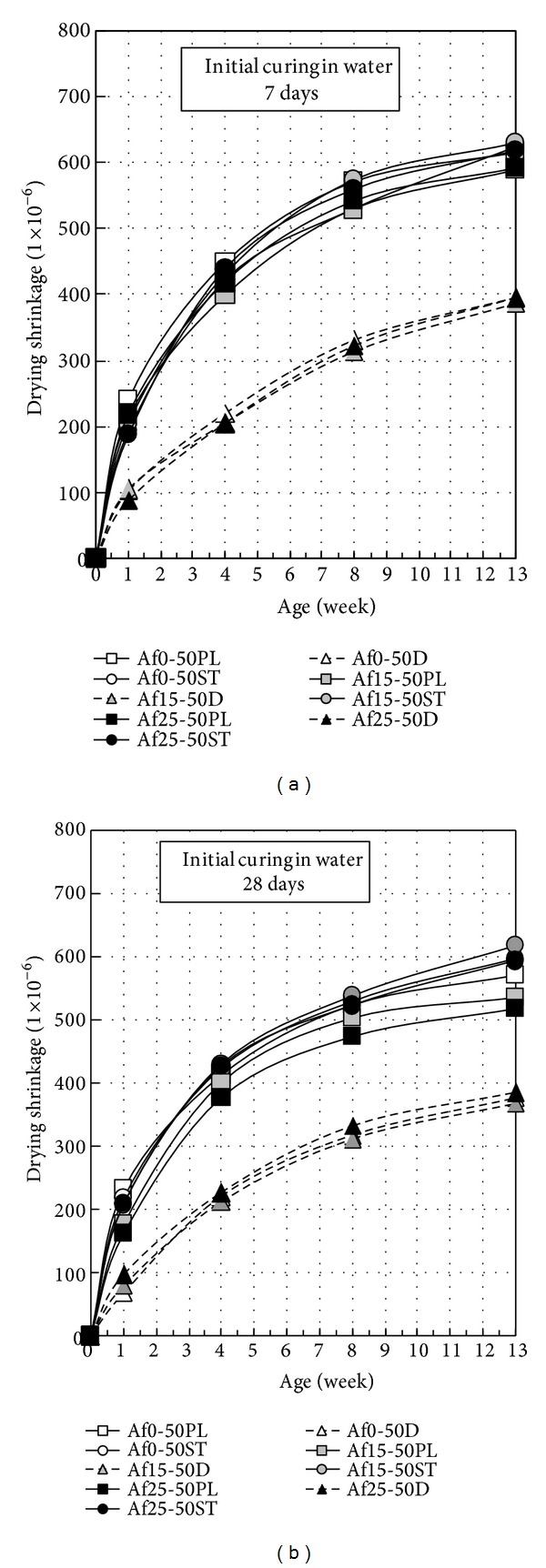
Change in length of concrete in 50% water-binder ratio.

**Figure 7:**
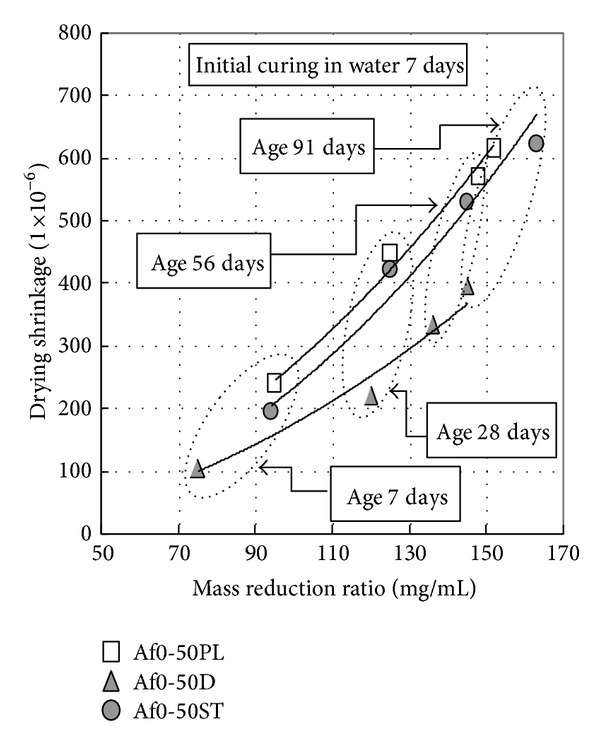
Relation between mass reduction ratio and drying shrinkage.

Upon Figures 5 and 6, drying shrinkage of nonair-entraining concrete (PL) is slightly less than air-entraining concrete (ST), because of differences in air content. From Figure 6, it is also observed that drying shrinkage decreases slightly with the increase of precuring period. On the other hand, when durability improving admixture is used, a significant effect is confirmed in reducing drying shrinkage. Compared to those materials where durability improving admixture is not used, it is possible to suppress change in length by around 60% using durability improving admixture [21].  Figure 7 shows the relation between mass loss and drying shrinkage. There is a clear difference of drying shrinkage in the same mass loss; drying shrinkage of D concrete is much less than PL and ST concrete. It is considered that glycol ether derivative as the main ingredient of durability improving admixture reduces surface tension of the capillary void water, lowers capillary tension, and then greatly reduces drying shrinkage. Furthermore, the reason that mass loss of D concrete is smaller than other concretes in the early age can be considered that most of glycol ether derivative is left in concrete (since only 1% of glycol ether derivative is soluble in water) to prevent capillary void water drying. As can be seen from Figure 15 on void distribution of concrete, the durability improving admixture itself remains in the capillary and gel voids within the concrete. It is assumed that drying shrinkage is reduced by glycol ether derivative suffusing those pores of 10 nm or less that have especially large capillary tension.

### 3.4. Carbonation Acceleration Experiment

By means of conventional carbonation rate method, the carbonation depth is expressed by (1) given below [22]. Consider

(1) $$\begin{array}{c} C=A\sqrt{t}, \end{array}$$

where *C*: carbonation depth, mm, *t*: elapsed time, week, and *A*: carbonation rate, mm/$\sqrt{\text{week}}$.

**Figure 8:**
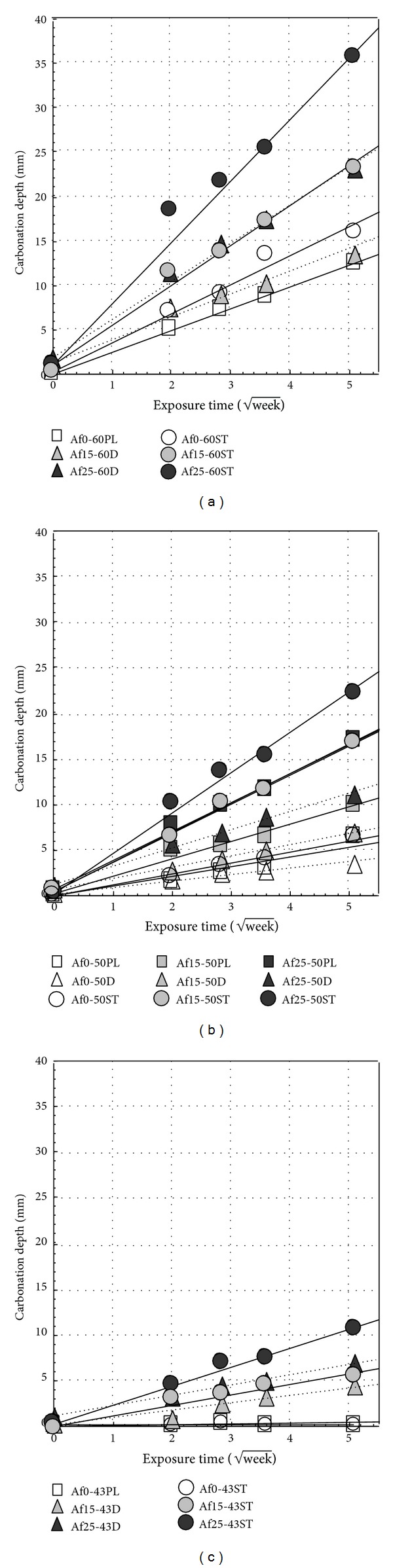
Carbonation depth by accelerating carbonation (at 5% CO_2_).

Figure 8 shows the results of carbonation depth by accelerating carbonation test. Carbonation depth is found to be increased with mixing fly ash and decreased with the decreases of water-binder ratio. Moreover, in the case of using ordinary cement, the carbonation depth of air-entraining concrete is slightly larger than nonair-entraining concrete [23]. However, this experiment results show that carbonation depth of nonair-entraining concrete is far less than air-entraining concrete regardless of water-binder ratio. This can be considered that the concrete structure is more compact due to nonair-entraining capability and then inhibited the carbonization.

**Figure 9:**
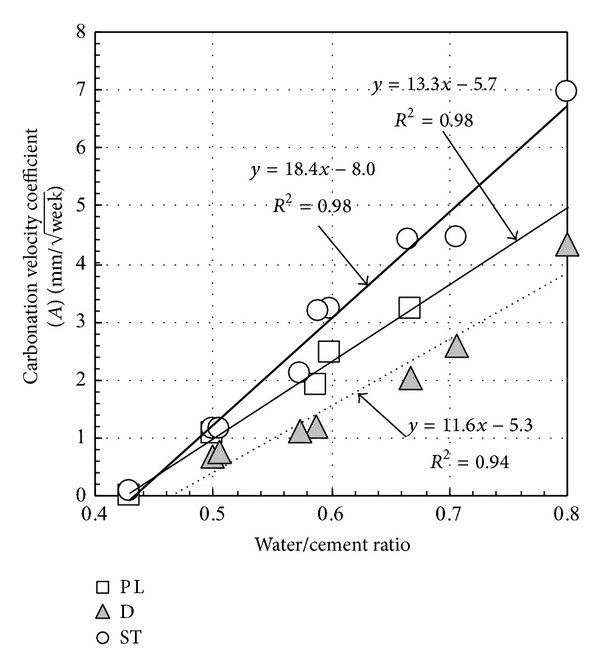
Relation between water-cement ratio and carbonation velocity coefficient (initial curing methods I).

Figure 9 shows the relation between water-cement ratio and carbonation velocity coefficient which derived from linear regression of Figure 8. The carbonation velocity coefficient is expressed by (2) given below. Consider

(2) $$\begin{array}{c} x=A\sqrt{t}+B, \end{array}$$

where *x*: carbonation velocity coefficient; *A*: carbonation rate, mm/$\sqrt{\text{week}}$; *B*: carbonation depth at the start of carbonation acceleration, mm.

After 4 weeks of curing in water and 4 weeks of curing in air, because the self-carbonation caused by pozzolanic reaction of fly ash is negligible, it is considered that carbonation velocity coefficient is roughly equal in the same water-cement ratio regardless of fly ash replacement ratio. The ratio of carbonation velocity coefficient of fly ash concrete is ST : PL : D = 18.2 : 13.3 : 11.6 = 1.0 : 0.7 : 0.6. It indicates that nonair-entraining mode can significantly reduce the carbonation rate.

**Figure 10:**
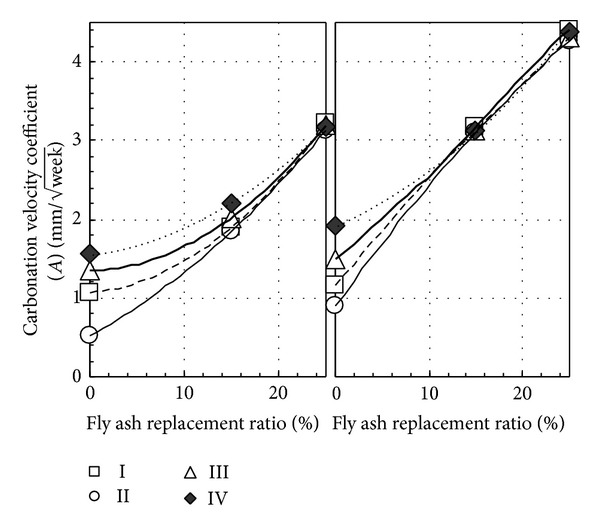
Relationship between fly ash replacement ratio of different precuring pattern and carbonation velocity coefficient.

The carbonation acceleration experiment is an experiment in raising the carbon dioxide concentration in the surrounding atmosphere, which accelerated carbonation of the concrete and evaluated anticarbonation ability by measuring carbonation depth. In this experiment, 4 different types of precuring carbonation acceleration experiment were applied for water-binder ratio of 50%. Among them, there are initial curing methods I: after 28 days of curing in water then curing in air for 28 days, initial curing methods II: after 28 days of curing in water then curing in seal for 63 days, and finally curing in air for 28 days; initial curing methods III: after 28 days of curing in water then curing in air for 91 days; and initial curing methods IV: after 7 days of curing in water then curing in air for 91 days.  Figure 10 shows the relationship between fly ash replacement ratio of different precuring pattern and carbonation velocity coefficient. From this figure, owing to the different precuring mode, carbonation velocity coefficients of PL and ST concrete without adding fly ash were largely affected by precuring, unrelated to the air content. However, carbonation velocity coefficient with fly ash replacement cement reached the same level unrelated to precuring method and period. In other words, for more than 15% of fly ash replacement ratio in concrete, it can be considered that larger carbonation velocity coefficient was affected by self-carbonation of fly ash compared to influence of precuring. From the above results, carbonation acceleration experiment of concrete with more than 15% fly ash replacement ratio depends on the carbonation depth at the start.

### 3.5. Freezing-Thawing Resistance

In this experiment, 3 different types of precuring freezing-thawing experiments were applied. Among them, there are initial curing methods A: curing in air for 28 days; initial curing methods B: after 28 days of curing in water then curing in air for 14 days; and initial curing methods C: after 28 days of curing in water then curing in air for 28 days.

**Figure 11:**
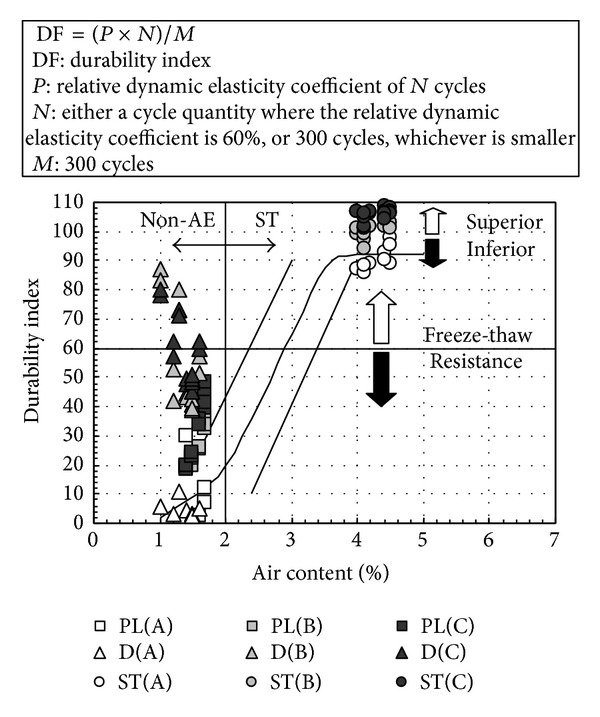
Relation between air content after hardening and durability index.

**Figure 12:**
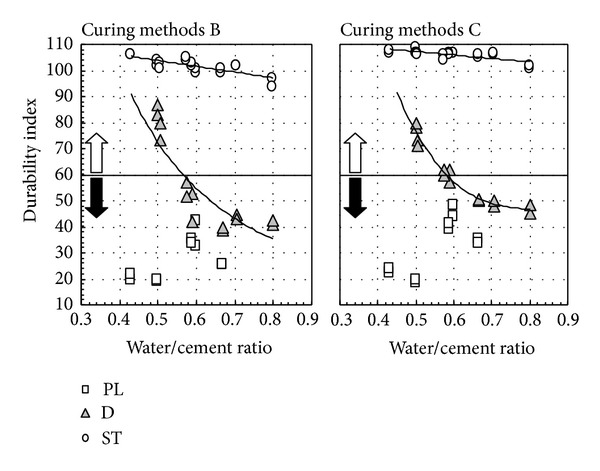
Relationship between water-cement ratio and durability index.

Figure 11 shows the relation between air content after hardening and the durability index, and Figure 12 shows the relationship between water-cement ratio and the durability index. As shown in Figure 11, regardless of water-binder ratio and fly ash replacement ratio, ST concrete shows favorable freezing-thawing resistance. On the other hand, in this experiment, the durability index of nonair-entraining concrete is below 40% with 4 weeks of water immersion in the precuring stage. However, when durability improving admixture is used, freezing-thawing resistance improved substantially by conducting the drying process for 2 weeks in the precuring stage even in nonair-entraining concrete, regardless of water-binder ratio. In addition, from Figure 12 it can be observed that even the drying process is conducted for PL concrete, and freezing-thawing resistance will not be improved. However, to ensure the water-cement ratio of 60%, freezing-thawing index of concrete with durability improving admixture used can reach above 60%, regardless of the length of the drying process. Considering the curing period in actual construction work which is very short and concrete is stripped after casting and exposed in air, fly ash concrete can be durable to freezing-thawing with the use of durability improving admixture and ensure the proper water-cement ratio, even when using nonair-entraining type [24].

### 3.6. Characteristics of Air Bubbles

**Figure 13:**
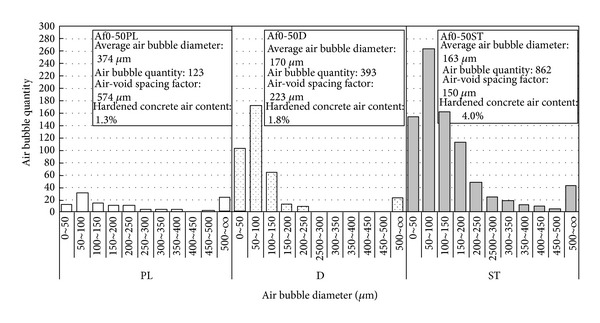
Relationship between air bubble diameters and quantity of air bubbles.

**Figure 14:**
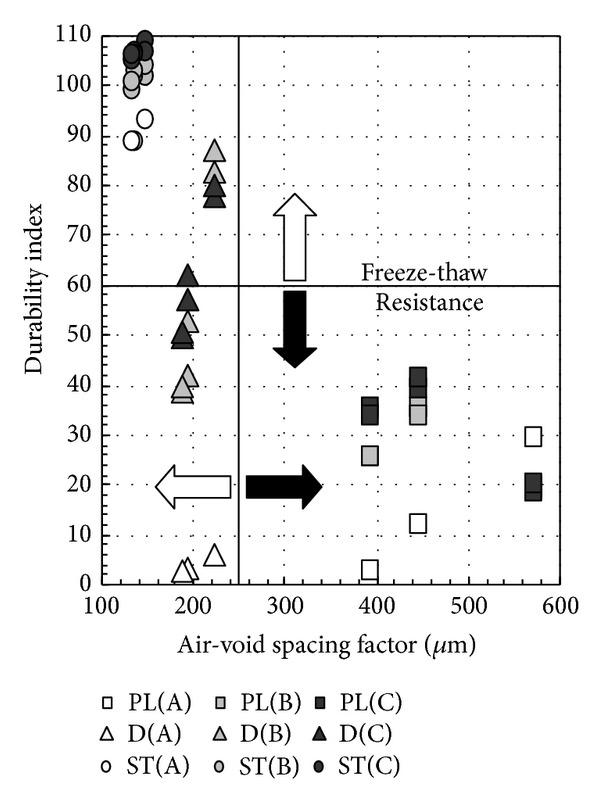
Relationship between air void spacing factor and durability index.

Figure 13 shows the relationship between air bubble diameters and quantity of air bubbles, and Figure 14 shows the relationship between the air void spacing factor and the durability index. It is clear that the number of air bubbles is greater and the average diameter is smaller with durability improving admixture used, compared to PL concrete. The influence of mixing in fly ash is that there is a tendency for the number of air bubbles to increase and the air void spacing factor decreased with the increase of fly ash replacement ratio, without regard to the amount of air or the use of durability improving admixture. The air void spacing factor with durability improving admixture used is lower than 200~250 *μ*m of antifreeze damage index as shown in Figure 14. In addition, there is a strong defoaming reaction of durability improving admixture, which can drive out coarse air bubbles over 500 *μ*m. The air content of fresh concrete is the same in PL and D concrete, but the content of air in D concrete becomes 0.5~1.0% greater. This is considered that the glycol ether derivative of the durability improving admixture exists in hardened concrete in the form of tiny droplets under 150 *μ*m. Here, the mechanism of durability improvement is discussed in the perspective of air bubble system. By 2-week drying process in precuring, resistance to freeze damage is improved significantly. When void water dried, a part of glycol ether derivative in the concrete is taken into capillary and gel air cavities, and the original oil specks perform the same function as air bubbles, easing water freeze expansion. It is thought that this improves the resistance to freeze damage.

**Figure 15:**
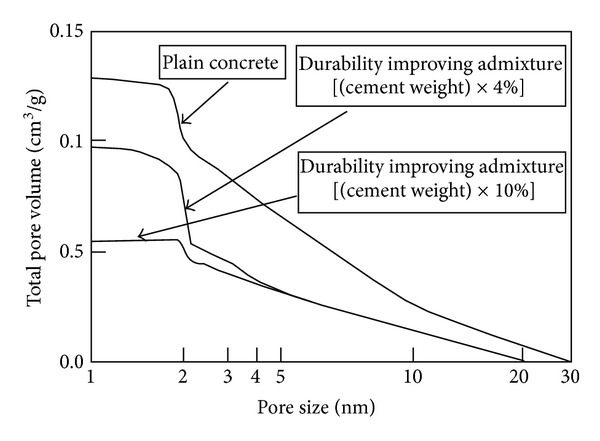
Concrete cavity distribution when durability improving admixture is used.

**Figure 16:**
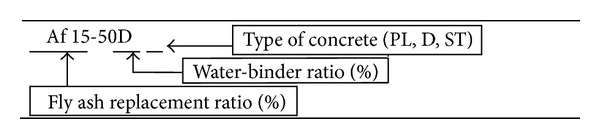
Interpretation of marks.

Concerning the effect of durability improving admixture in preventing freeze damage, a significant improvement is noted by 2-week drying process in precuring. Considering cavity distribution in concrete, when durability improvement admixture is used, as shown in Figure 15, by its strong bubble elimination action, the admixture reduces the entrapped air and replaces it with the same amount of entrained air bearing 250 *μ*m or less of oil specks into the hardened concrete. Furthermore, accompanying the drying of void water, by injecting a portion into capillary cavities, shrinkage is lowered. The same reaction occurs in gel cavities, which are the lamellar cavities formed in lamellar crystal while C-S-H hydrate is produced. It is believed that hardened concrete is formed and durability is improved through these processes. Figure 15 shows concrete cavity distribution when durability improving admixture is used.

## 4. Conclusions

The following conclusions were drawn from the work presented above on the effects of durability improving admixture, air content, water-binder ratio, and fly ash replacement ratio on fly ash concrete properties.

1. By using durability improving admixture in nonair-entraining fly ash concrete, the compressive strength of fly ash concrete can be improved by 10%–20%, and its initial compressive strength improved also.
2. Irrespective of the presence of air, durability improving admixture, or fly ash replacement ratio, both tensile strength and modulus of elasticity are dependent on compressive strength.
3. By using durability improving admixture in fly ash concrete, the drying shrinkage is reduced by 60%.
4. Carbonation resistance of concrete is roughly proportional to water-cement ratio regardless of water-binder ratio and fly ash replacement ratio. The ratio of carbonation velocity coefficient of fly ash concrete is ST : PL : D = 1.0 : 0.7 : 0.6. Also, carbonation acceleration experiment of concrete with more than 15% fly ash replacement ratio depends on the carbonation depth at the start.
5. By using durability improving admixture for 2 weeks of curing in air (drying process), the freezing-thawing resistance can be improved even in nonair-entraining concrete.

By making use of durability improving admixture, it is easier to control the air content and make fly ash concrete into nonair-entraining one. The quality of fly ash concrete is thereby optimized. In the field of application fly ash concrete, the use of nonair-entraining mode and durability improving admixture is a valuable option.

## Figures and Tables

**Table 1:** Characteristics of materials used.

Cement	Ordinary Portland cement Density: 3.16 g/cm^3^; specific surface area: 3280 cm^2^/g
Binder	Fly ash
Fine aggregate	Mountain sand Density in oven-dried condition: 2.56 g/cm^3^; water absorption: 1.72%; fineness modulus: 2.67
Coarse aggregate	Crushed stone Density in oven-dried condition: 2.64 g/cm^3^; water absorption: 0.81%; maximum size: 20 mm
Admixture	Air-entraining and water reducing admixture Air-entraining admixture for fly ash: nonionic surface active agent Durability improving admixture: glycol ether derivative (nonwater solution)

**Table 2:** Properties of fly ash used.

Ignition loss [%]	Density [g/cm^3^]	Blaine's value [cm^2^/g]	MB absorption [mg/g]
1.4	2.24	4160	0.51

**Table 3:** Experiment factors and ratios.

Experiment factors	Levels
Fly ash replacement ratio	0%; 15%; 25%
Water-binder ratio	60%; 50%; 43%
Air content	1.5 ± 0.5%; 4.5 ± 0.5%
Durability improving admixture	0 kg/m^3^; 10 kg/m^3^

**Table 4:** Mixture proportion and properties of fresh and hardened concrete.

	W/B (%)	W/C (%)	FA/B (%)	Mixture proportion (kg/m^3^)						Fresh concrete		Compressive strength (N/mm^2^)		
				W	C	FA	S	G	D	Slump (cm)	Air (%)	7 days	28 days	91 days
Af0-60PL	60	60	0	182	303	0	856		—	19.0	1.7	26.0	35.5	41.0
Af0-60ST				170	283	0	813		—	18.1	4.0	25.3	36.1	38.7
Af15-60D		71	15	166	235	42	880	1014	10	18.6	1.4	20.7	29.2	40.6
Af15-60ST				166	235	42	815		—	18.6	4.2	17.6	27.7	35.8
Af25-60D		80	25	164	205	68	879		10	18.2	1.5	12.3	20.4	26.8
Af25-60ST				167	209	70	801		—	17.0	4.1	13.7	22.6	32.4

Af0-50PL	50	50	0	182	364	0	806		—	18.8	1.4	36.1	47.5	53.0
Af0-50D				174	348	0	814		10	18.7	1.0	35.8	46.4	54.1
Af0-50ST				170	340	0	766		—	18.7	4.4	33.3	45.5	50.4
Af15-50PL		59	15	174	296	52	823		—	18.5	1.7	30.3	43.2	55.6
Af15-50D				166	282	50	831	1014	10	18.2	1.2	28.6	38.3	51.4
Af15-50ST				166	282	50	766		—	18.2	4.5	25.0	36.5	45.8
Af25-50PL		67	25	172	258	86	820		—	18.5	1.6	26.3	34.5	51.4
Af25-50D				164	246	82	829		10	17.7	1.5	22.8	33.8	43.7
Af25-50ST				167	251	84	751		—	18.6	4.1	20.2	31.3	43.1

Af0-43PL	43	43	0	189	440	0	726		—	19.2	1.5	45.4	57.8	68.1
Af0-43ST				178	414	0	684		—	18.3	4.5	38.7	50.7	56.5
Af15-43D		51	15	173	342	60	752	1014	10	17.8	1.3	35.0	47.9	53.0
Af15-43ST				176	348	61	673		—	17.7	4.5	31.9	43.9	55.0
Af25-43D		57	25	171	298	99	748		10	17.8	1.6	30.0	42.6	55.3
Af25-43ST				177	309	103	655		—	18.3	4.4	26.6	37.6	48.6

Notes: W/B, W/C, and FA/B are, respectively, means of water-binder ratio, water-cement ratio, and fly ash binder ratio. W, C, FA, S, G, and D are, respectively, means of water, cement, fly ash, fine aggregate, coarse aggregate, and durability improving admixture.
